# Supplementary material for: TIGD1 Function as a Potential Cuproptosis Regulator Following a Novel Cuproptosis-Related Gene Risk Signature in Colorectal Cancer
Source: Cancers (Basel). 2023 Apr 13;15(8):2286. doi: 10.3390/cancers15082286 (PMC10137011; doi:10.3390/cancers15082286)
Supplement: Supplementary file 1 [file cancers-15-02286-s001.zip › cancers-2302441-supplementary.pdf]

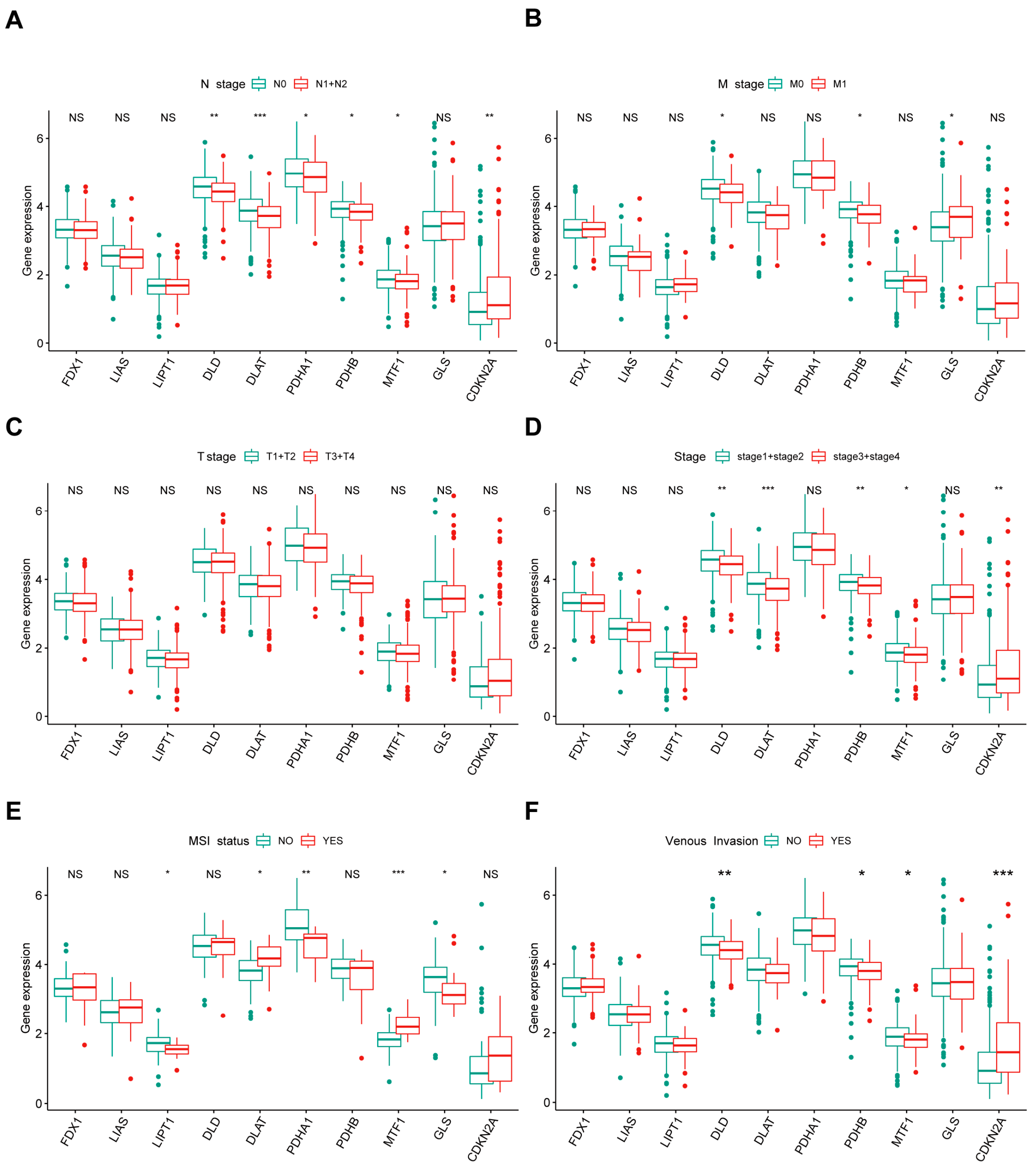

Supplementary Figure 1 | Ten cuproptosis markers correlated with clinicopathological features in the TCGA database; (A) correlation between the ten cuproptosis markers and N stage; (B) correlation between the ten cuproptosis markers and M stage. (C) correlation between the ten cuproptosis markers and T stage; (D) correlation between the ten cuproptosis markers and tumor stage; (E) correlation between the ten cuproptosis markers and microsatellite status; (F) correlation between the ten cuproptosis markers and venous invasion condition; *p < 0.05, **p < 0.01, and ***p < 0.001. ns, no significance

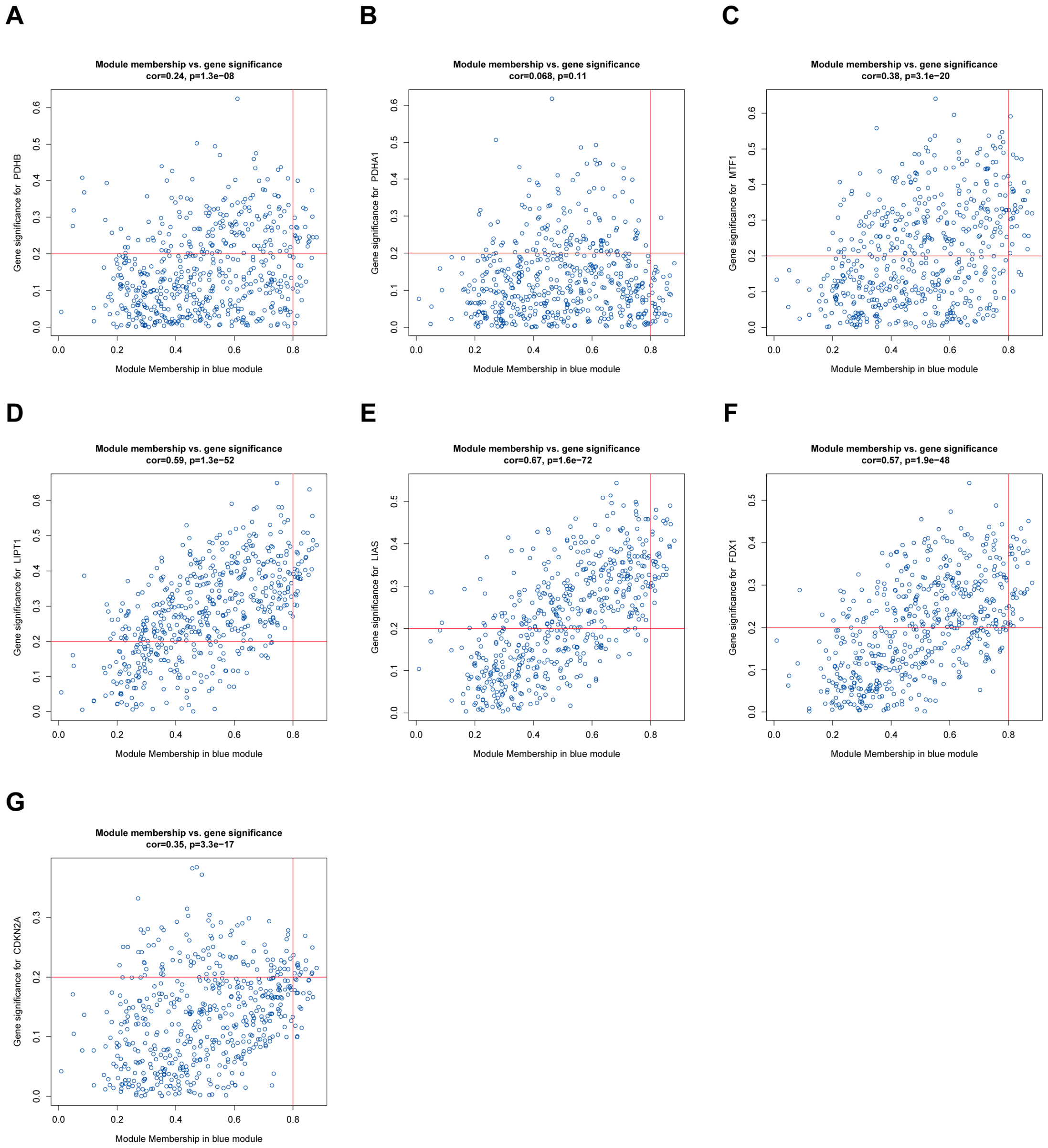

Supplementary Figure 2 | (A–G) Scatterplots of gene significance for cuproptosis markers (PDHB, PDHA1, MTF1, LIPT1, LIAS, FDX1, and CDKN2A) vs. module members in the blue modules (each dot represents a gene)

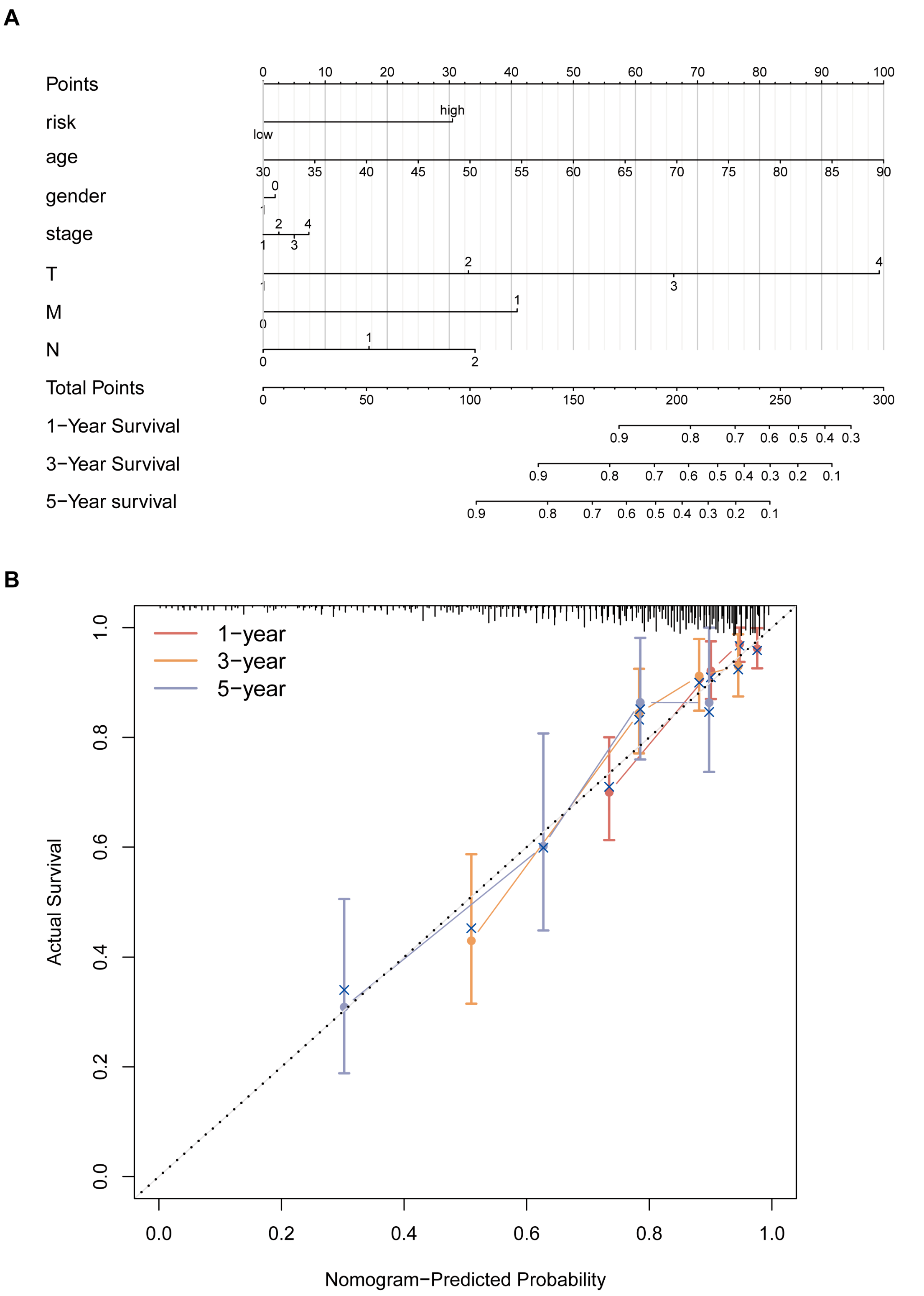

Supplementary Figure 3 | (A) Nomogram to predict the 1-year, 3-year, and 5-year OS rates of patients with CRC; (B) calibration curve for evaluating the accuracy of the nomogram model. The dashed diagonal line in black represents the ideal nomogram.

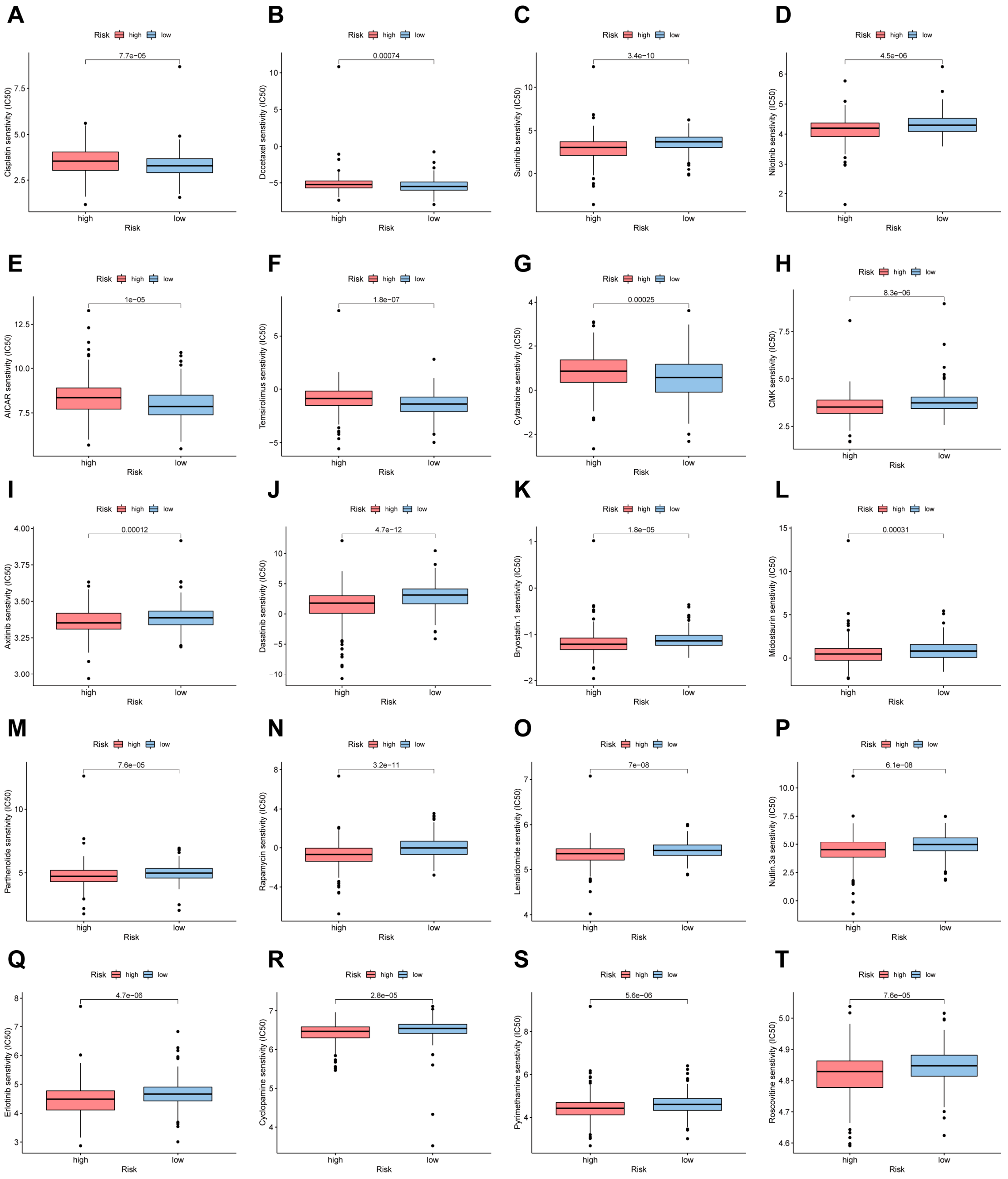

Supplementary Figure 4 | (A–T) Boxplots demonstrating the mean differences in estimated IC_{50} values of 20 representative drugs (cisplatin, docetaxel, sunitinib, nilotinib, AICAR, temsirolimus, cytarabine, CMK, axitinib, dasatinib, bryostatin.1, midostaurin, parthenolide, rapamycin, lenalidomide, nutlin.3a, erlotinib, cyclopamine, pyrimethamine, and roscovitine) between the two risk groups

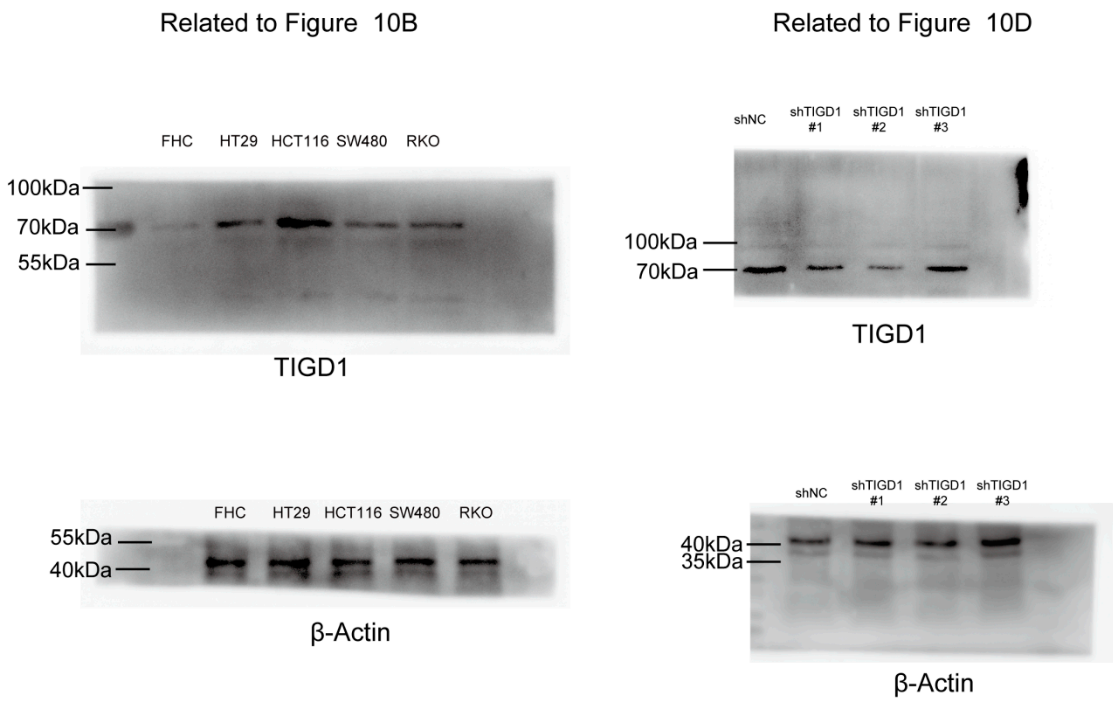

Supplementary Figure 5 | Uncropped Western Blots for Figure 10B and Figure 10D.

Supplementary Table 1 | Primer sequence for qRT-PCR

Primer sequence for qRT-PCR	
ASPHD1-F	GGATGGGTAGAGTGAGGCG
ASPHD1-R	AGGCAGGTCTGGTAGGAAAAG
PPP1R13L-F	TGCAACGACACAGTCATCTG
PPP1R13L-R	GCCCCATACTCTGCTCGAC
SUCLG2-F	CAAAAGACCCTAATGTTGTGGGA
SUCLG2-R	TTCAGCAACCATCACCTTGTT
KLHL35-F	ATGGACCTAGCTGAAGTGATCG
KLHL35-R	CTCTCTGGATGGTAGGCATCG
TIGD1-F	TGCCCCTAGTCATCCAAGAG
TIGD1-R	TGTTGTGTTAGCAGGCATGAAA
PMAIP1-F	ACCAAGCCGGATTTGCGATT
PMAIP1-R	ACTTGCACTTGTTCCTCGTGG
DMPK-F	TGGCGGAGATTGTCATGGC
DMPK-R	GGATGTTGTCGGGTTTGATGTC
GAPDH-F	GGAGCGAGATCCCTCCAAAAT
GAPDH-R	GGCTGTTGTCATACTTCTCATGG

Supplementary Table 2 | Gene symbols in the blue module identified using WGCNA

Gene symbols in blue module identified by WGCNA							
SLC66A2	SLC7A11	ERCC6L	SLC2A12	CHRNA5	PRC1	ZNF239	NFE2L3
ACAT1	TRMT6	HSD17B11	LGR6	MCM8	CCNA2	MZT1	EPHX4
KIF24	PAICS	LGR4	PDE9A	IMPA1	HS6ST2	CTPS1	CIP2A
CENPU	VSIG2	RCBTB1	GTF3A	PUS7	CHEK1	RMDN2	VILL
MACIR	CLCN5	HASPIN	EDN3	ME1	PTPRH	ETHE1	PCLAF
BEST4	JSRP1	MTERF3	RASD2	RAVER2	APOBR	NUP62CL	MGLL
SOX4	TMEM67	CDK20	ENTPD8	FIGNL1	RRM2	KIF23	C2orf15
STIL	PHKA1	PLEKHN1	RFC3	CENPE	GJC2	E2F5	INTS13
NECTIN3	NUSAP1	DNA2	OSBPL3	TBC1D31	FCGBP	POP1	SQOR
ATAD5	KITLG	ABCE1	KIF14	EXOSC8	SDCBP2	KLK12	ENOPH1
FANCI	KIAA1549	GNA13	TEX30	SINHCAF	KCNH8	DBF4	MELK
GRAMD2B	SKA1	KIF4A	LRRC6	SUCLG2	ABHD5	KPNA2	ARHGEF38
GABARAP	TOM1L2	DDX55	AUNIP	SLC25A42	CDC7	FANCD2	PIP5K1B
THUMPD2	NUP58	XPO4	TMEM97	NCOA4	TGIF1	PLCH2	AP3S2
UBE2C	KRCC1	CDC6	ZNF367	MTFR2	RIPK2	DDX21	KCTD9
ASPHD1	MRPS23	SH3BGRL2	NAA25	MB	PAQR5	TGDS	MET
ZNF850	KIAA0895	C1orf115	SQLE	CGAS	SOD3	LGALS9C	PROSER1
DIAPH3	MMS22L	PDCD5	AURKA	RNASE1	LYPD8	GRIN2D	RASAL1
TBC1D30	MAD2L1	RAP1A	ZNF280C	LRP8	RACGAP1	MIER3	SLC7A6
ECT2	PDP1	NANP	PLXNA2	PRDX6	NFXL1	HSPA4L	MORC4
WDR75	TTK	NUF2	LTBP4	TRIM59	ERO1A	A1CF	WASL
NECTIN4	ZGRF1	SERINC2	NOLC1	RPL22L1	SYTL1	ENC1	CLDN1
SDHD	TDP2	PPIL1	RBM28	CENPI	SMIM14	NCAPG2	SGO1
ATAD2	RTN4R	RAC3	UGDH	JADE3	TNFRSF10B	SCP2	CEP55
LDHD	CLIC3	POLD4	LYAR	CASD1	GINS4	NEURL1	ZER1
DUS4L	TAF1D	LIPG	BRCA2	GINS1	PDK2	PRSS22	GDPD2
RHOF	RBL1	GPD1L	FIBCD1	BRCA1	GRAMD1C	TP53I3	DMPK
FGFRL1	HSPD1	GPSM2	EXO1	CDA	KLK1	TSEN2	HMGA2
COL17A1	MTM1	POLR1D	ACVRL1	TXLNG	ZFYVE28	NOP58	PRR11
RCL1	MXI1	B3GALT4	UGP2	TRAF5	HOMER1	LRRC37A3	HEATR1
COCH	CYP39A1	WDR43	C1orf112	ECI2	RAD54B	ETFDH	MALL
B3GNT6	NOC3L	IFITM2	CCSAP	RPP40	KIF18A	TFF1	OTUD6B
MSH5	NCR3LG1	UGGT2	CLSPN	XPNPEP3	NAP1L1	SMPD1	GARS1
CDKN3	SMOX	HSD17B2	P2RX4	PLS1	GRPEL2	HADHB	POLR1B
SLC6A8	PDE4C	PCMTD2	PACSIN2	RBP4	GOLGA8B	SCML1	PLCD3
HMMR	STRIP2	DCAF13	EBPL	SCLY	DDIAS	MINDY1	CD46
COL9A2	E2F7	TAF1A	SLC25A32	CENPF	MTHFD2	CCT6A	TMEM41A

ZNF485	ORC6	PLCD1	UCHL3	DDX31	TMEM59	CCNJL	SHCBP1
CENPK	ZNF117	IARS1	POLR3G	MIER1	SPPL2A	TEX10	CENPA
C19orf33	ADAMTSL5	ACE	ZNF473	SETD6	APPL2	PNPT1	BRIX1
NEIL3	PCSK6	ANLN	DCUN1D5	ACADS	LRP11	CMSS1	ATP2A3
SKP2	CBX3	URB2	AP3M2	CCNB1IP1	FAM111B	POLQ	SLC39A10
AP1S3	PRMT3	MMP28	CDC14A	PPARGC1B	TOP2A	KNSTRN	INAVA
ANXA3	MKI67	WDHD1	MSLN	NIPAL1	ARMC10	KLHL35	DDX10
PACC1	LGR5	NCAPD3	DNAJC2	PBK	HPN	KNTC1	GNL3
MND1	SPC25	SESN2	SLC9A1	SCAMP2	HELLS	TIGD1	TLCD4
GOLGA7B	SUV39H2	MSI2	PDE8A	CRAT	PSPH	PFDN4	KIF20B
ARNTL2	EHHADH	PGM1	TARBP1	PPARD	CDCA7	FXYD5	RILP
TTC26	FBLIM1	PINK1	IPO5	ACADM	FZD3	NIFK	ZNF251
PPM1H	SNTB1	NEK2	SMYD3	TPSG1	DONSON	WDR12	MACC1
ASPM	MAGOHB	PAN3	CENPH	PLCE1	SLC25A27	UBE3D	SLC12A2
NUP155	RHBDF2	EEF1E1	CNN2	PRKDC	SLC25A29	CENPP	PSMG1
RETSAT	ALPI	BLM	STOX1	CSE1L	ABCD3	TSPAN1	TRIM29
POLE2	SERPINF2	CCNYL1	ITM2C	CHD7	PUM3	NPM1	RNF32
SEZ6L2	PNO1	TXNRD3	PERP	SCD	DKC1	BRIP1	LRRC1
ATP6V1E2	MELTF	SLC35G1	NKRF	GPRIN2	HSPH1	CDK1	RPGRIP1L
BUB1	RAD51AP1	C5orf34	RTKN2	SPIN4	ADAT2	HAUS6	DGKH
NUDCD1	BUB1B	SKA3	DEPDC1B	PADI2	HIGD1A	SEMA3F	SPDL1
PRSS2	RETREG1	MCOLN2	VSNL1	PCID2	GINS3	CIPC	GNG12
ZC3H8	RCN1	ACVR1C	SMTN	SLC7A1	NEBL	CIT	ZDHHC9
PPP1R13L	EEF1AKMT1	CDKN1A	PROX1	PPAT	XPOT	TPX2	FMNL2
CBFB	PMAIP1	BCAS1	XRCC2	DSN1	STK17B	URB1	HACD3
DTL	SLC9A7	PDCD4	JPH1	NCAPG	CDK2	POLB	ZNF575
GTPBP4	CKAP2	PARPBP	ZC3HAV1L	GPR180	ETNK1	MTHFD1L	ZNF121
CKAP2L	XPO5	EIF2S2	FAM169A	CDCA2	GOLGA8A	GTF2F2	TAX1BP3
RFC4	PRR36	MTBP	MPP6	GGCT	OCEL1	PPA1	CENPN
CHPF	DSCC1	PTGR1	ATP11A	UTP14A	VMA21	MGAT4A	ERV3-1
RAD18	CENPJ	AADAT	EZH2	IL10RB	AGRN	TTYH3	
DLGAP5	NUFIP1	PLK4	MCM10				
